# Meteoropathy: a review on the current state of knowledge

**DOI:** 10.25122/jml-2023-0097

**Published:** 2023-06

**Authors:** Malvina Hoxha, Bruno Zappacosta

**Affiliations:** 1 Department for Chemical - Toxicological and Pharmacological Evaluation of Drugs, Catholic University Our Lady of Good Counsel, Tirana, Albania

**Keywords:** meteoropathy, weather, pain, climate change, symptoms

## Abstract

Meteoropathy is no longer considered a popular myth, but a new disease that significantly impacts daily life, particularly in individuals who experience mental illness, cardiovascular disorders, and respiratory conditions. However, there are very limited data on this condition. This study aimed to comprehensively review and analyze existing in vivo animal studies and human clinical trials investigating the effects of meteoropathy on health and its pharmacological treatment. A thorough literature search was conducted across databases such as PubMed and Scopus to gather relevant information. Our analysis primarily focused on the relationship between meteoropathy and mental health, including the influence on affective temperaments. Additionally, we explored various treatment approaches, emphasizing the combination of muscle exercises, pharmacological interventions, and naturopathy, which have shown promise in alleviating pain among individuals affected by meteoropathy. Future research in meteoropathy should shed light on synthesizing new pharmacological compounds.

## INTRODUCTION

Meteoropathy, also considered a syndrome or a new disease, is known to affect our psychological and physiological health [1, 2]. The term originates from Greek, combining “meteora” (celestial phenomena) and “pathos”(pain), and was first introduced in the late 20th and the 21^st^ century [1, 3]. Studies show that almost 30 % of the world's population experiences some sort of meteoropathy [4]. Among these individuals, women, particularly menopausal women, are more prone than men to have meteoropathy [5, 6]. Moreover, the prevalence of meteoropathy is rising [7], attributed to the difficulties individuals face in adapting to weather fluctuations. This inadequate response of self-regulation mechanisms to such changes is thought to be influenced by the presence of electromagnetic waves encompassing a wide range of amplitudes and frequencies [3]. This is thought to be caused by modern lifestyle patterns, characterized by prolonged periods in closed air-conditioned environments that are either excessively cooled or heated [8].

Nevertheless, how do we define a meteoropath? There is a distinction between “meteoropathy” and “meteorosensitivity”. The former refers to individuals who develop new diseases or experience exacerbation of existing symptoms due to weather changes, while the latter encompasses individuals who are sensitive to weather changes on a physical and mental level [1]. The METEO-Questionnaire (METEO-Q) is used to detect meteorosentivity and meteoropathy. The questionnaire displays 11 items, each assigned a score rating from 0 (absent) to 4 (severe), classified into two major groups. Items 1-5 assess mood variations in relation to temperature, seasons, atmospheric changes, geographic zone, jet lag, and latitude. Items 6-11 assess the qualitative effects of symptoms (the interference with daily activities, their relationship with other cyclical phenomena, the propensity of the symptoms to minimize or disappear when the triggering condition stops, or when an opposite environmental condition shows up, etc.) [1]. The meteoropathy checklist includes twenty-one symptoms rated on a scale from 0 (absent) to 4 (severe). In a study by Mazza *et al*., different METEO-Q total scores were reported for meteoropathy and meteorosensitivity in women and men, respectively (meteoropathy: women: >11 versus > 9 in men; meteorosensitivity: women: >8 versus > 6 in men) [1]. Not all individuals with meteorosensitivity can develop the symptoms of meteoropathy [3].

There are two types of meteoropathy, primary and secondary, respectively. The primary form affects healthy people who complain of muscle and joint pain, mood changes, and physical weaknesses [4, 9, 10]. These symptoms fade away as soon as the weather is stabilized. On the other hand, the secondary form affects patients with chronic diseases, such as cardiovascular and obstructive pulmonary disease [11-14].

Meteorological variables, such as barometric pressure, air mass, temperature, humidity, cloudiness, weather fronts, wind speed, precipitation, and sunlight, affect health and are associated with changes in the concentration of cerebral neurotransmitters in the brain.

For instance, meteoropathic patients often exhibit elevated levels of adrenocorticotropic hormone (ACTH) produced by the pituitary gland, leading to symptoms such as palpitations, anxiety, and irritability. Conversely, endorphins, known as "happy hormones," are reduced, decreasing the pain threshold [15-19].

Another hypothesis suggests that the vagus nerve plays a role in meteoropathy. Recent research by Liebell *et al*. demonstrated that auricular acupuncture reduced weather-induced symptoms in humans [20]. When there are no indications of a thunderstorm that may be thousands of kilometers away, individuals with meteoropathy can sense it through the electromagnetic field [21].

Certain neurons in the superior vestibular nucleus respond to changes in barometric pressure in mice, which can also act similarly in humans [22]. When the atmospheric pressure drops, increased pressure in the inflamed tissues intensifies general pain and sinus headache [23, 24]. People extremely sensitive to weather experience changes in blood pressure and heart rate, stomach disorders, breathing difficulty, depression (mental and physical), numbness, anxiety, irritability, headaches, sleep disorders, increased vulnerability to pain in the joints, muscles pain, pain in head, neck and shoulders, dizziness, chronic pain, the desire to remain indoors [1, 3, 25, 26]. When exposed to low temperatures caused by air conditioning, blood vessels contract to reduce body temperature, especially indoors, compared to outdoors [27].

Symptoms typically diminish when the weather changes and may return with varying intensity during subsequent changes [1, 28]. The intensity of the symptoms increases before or after the weather changes rather than during the change itself. High humidity (>70%) and temperatures >30°C can lead to increased sweating and fluid loss as the body attempts to cool down [24, 29-31].

## METEOROPATHY IN ANIMAL MODELS

We identified only two experimental animal studies, both carried out in mice models, reporting information on meteoropathy (Table 1). Sato *et al*. observed differences in brain mechanisms responsible for emotions, specifically in the hypothalamus and amygdala nucleus, which can contribute to meteoropathy development in mice and potentially in humans [22]. C-Fos protein found in the superior vestibular nucleus was used as a marker of neural activation in mice placed in a climatic chamber where the barometric pressure was lowered [22]. The electromagnetic waves directly affected the hypothalamus, stimulating adrenocorticotropic hormone production and decreasing endorphin production, which enhanced meteoropathic symptoms [22]. In a recent study, Kurauchi *et al*. confirmed that female mice are highly suitable as meteoropathy animal models [32].

## EVIDENCE FROM HUMAN CLINICAL TRIALS

Table 1 summarizes the main characteristics of the nine human clinical studies that specifically assessed meteoropathy, excluding reviews and focusing solely on meteoropathy rather than meteorosensitivity. Most studies reported data on the implication of meteoropathy on the nervous system. Increasing evidence indicates that meteoropathy may affect mental health, leading to aggressive behavior, depression, sleep disorders, fear, and suicide [9]. Bellini *et al*. showed that the intensity of meteoropathy is correlated with levels of depression (r=.253; p< 0.01) and that patients with medication-overuse headaches would most likely experience higher intensity and quantitative levels of meteoropathy [33]. Moreover, meteoropathy has been linked to endocrinological and neurophysiological variations and social and emotional aspects of patients' lives [33]. Licanin *et al*. showed a higher admission rate of individuals with specific psychiatric disorders associated with certain seasons and specific weather parameters [8]. Di Nicola *et al*. revealed that METEO-Q scores were directly correlated with suicide attempts in bipolar patients [9].

**Table 1:** Summary of the main outcomes of all studies on meteoropathy (reviews excluded)

Study	Animal/Human models	Sample size and characteristics	Main outcomes
Bellini S, Migliorati M, Ricci F, Erbuto D, Pompili M. The association between meteoropathy, depression, hopelessness and quality of life in medication-overuse headache patients. J Headache Pain. 2015;16(Suppl 1):A50	Human	203 adult outpatients admitted to the local Headache Centre of the Sant’Andrea Hospital in Rome, Italy	Meteoropathy was correlated with endocrinological and neurophysiological variations and was associated with social and emotional relations, physical health and activities, and depression [33].
Kurauchi Y, Tanaka R, Ryu S, Haruta M, et al. Establishment of meteoropathy model mice. Proc 94th Annu Meet Jpn Pharmacol Soc. 2021.	Animal (Mice model)	N.S	Female mice represented a highly valid meteoropathy animal model [32].
Fujii T, Saito S, Yamasaki N, Fujii T. Weather changes leading to bleeding in arthropathic joints among individuals with haemophilia: Symptoms of meteoropathy? Haemophilia. 2020;26.	Human	24 patients with haemophilia	When the weather changed, some patients with haemophilia had increased bleeding in arthropathic joints [34].
Loth FL, Liebensteiner MC, Giesinger JM, Giesinger K, Bliem HR, Holzner B. What makes patients aware of their artificial knee joint? BMC Musculoskelet Disord. 2018;19(1):5.	Human	40 patients with total knee arthroplasty	7 patients (18%) reported that joint awareness was associated with meteoropathy [35].
Di Nicola M, Mazza M, Panaccione I, Moccia L, et al. Sensitivity to Climate and Weather Changes in Euthymic Bipolar Subjects: Association With Suicide Attempts. Front Psychiatry. 2020;11:95.	Human	352 euthymic bipolar disorder patients v.s 352 healthy control subjects	METEO-Q scores were directly correlated to suicide attempts in bipolar patients [9].
Licanin I, Fisekovic S, Babić S. Admission rate of patients with most common psychiatric disorders in relation to seasons and climatic factors during 2010/2011. Mater Sociomed. 2012;24(2):94-99.	Human	2355 respondents at the Psychiatric Clinic, Clinical Center of Sarajevo University	The results showed an increased admission rate of individuals with specific psychiatric disorders associated with certain seasons and weather parameters [8].
Rzeszutek M, Oniszczenko W, Zalewska I, Pięta M. Personality profiles and meteoropathy intensity: A comparative study between young and older adults. PLoS One. 2020;15(11):e0241817.	Human	758 participants (378 young adults (18–30 years old) and 380 older adults (60+ years old).	Not all individuals with meteorosensitivity had the same personality trait pattern [3].
Sato J, Inagaki H, Kusui M, Yokosuka M, Ushida T. Lowering barometric pressure induces neuronal activation in the superior vestibular nucleus in mice. PLoS One. 2019;14(1):e0211297.	Animal (mice)	34 (18 male vs. 16 female)	Certain neurons in the superior vestibular nucleus responded to changes in barometric pressure in mice, which can act similarly also in humans [22].
Oniszczenkow W. Affective Temperaments and Meteoropathy Among Women: A Cross-sectional Study. PLoS One. 2020;15(5):e0232725.	Human	450 Caucasian women	Asthenia was the most severe symptom of meteoropathy [7].
Terentjev K, Andronov S, Lobanov A, Popov A, et al. Method for predicting meteopathies in patients with arterial hypertension. Eur Heart J.2021;42(Supplement_1):ehab724-2322.	Human	24,494 requests	The risk of having a hypertensive crisis in patients with arterial hypertension associated with an increased risk of meteopathy could be predicted through logistic regression models [36].
Liebell D. Vagus Nerve Hypothesis: Regulatory Mechanism and Treatment Fluctuations in Atmospheric Barometric Pressure. Integr Complement Ther. 2022;28(4)	Human	N.K	Reduced weather-induced symptoms were observed following auricular acupuncture [20].

N.S-not specified; N.K-not known

Despite the small sample size, Fuji *et al*. observed that weather changes could lead to increased bleeding in arthropathic joints among patients with hemophilia, which could decrease when taking prophylactic treatment (factor VIII or IX concentrate) [34]. Loth *et al*. reported that joint awareness is also related to meteoropathy (18%) [35]. Recently, Terentjev *et al*. used climatic and geomagnetic conditions to develop a logistic regression model to calculate the risk of having a hypertensive crisis in patients with arterial hypertension. The data showed that in the summer, the risk was higher (89%), followed by autumn (77%), winter (77.3%), and spring (59%) period [36].

## POTENTIAL THERAPEUTIC AGENTS FOR METEOROPATHY

Exercise therapy, such as muscle stretching, strengthening, and endurance, can help reduce pain in meteoropathic patients that usually suffer from head, neck, and shoulder pain. The intensity and time of exercise should be decided by a physiotherapist based on the pain threshold [37]. Adopting a healthy lifestyle, including physical activity, a balanced diet rich in fresh fruits and vegetables, supplements containing magnesium and vitamin B complex, water consumption, normal circadian rhythms, and light therapy can help prevent pain. Fats and synthetic sweeteners should be avoided as they increase the digestive tract circulation. In regards to pharmacological treatment, there is no specific treatment for meteotorpathy. The presence of comorbidities could influence the treatment. Non-steroidal anti-inflammatory drugs (NSAIDs) and anti-vertigo drugs (dimenhydrinate, diphenidol, diphenhydramine) taken in advance were reported to be efficient [37].

Nonetheless, the potential side effects of commonly used drugs in meteoropathic patients, such as diuretics, angiotensin-converting enzyme inhibitors, angiotensin II receptor blockers, and psychoactive drugs, are still unknown [24, 38]. Since vasodilatation is a side effect of diuretics but also occurs in thermoregulation, diuretics should be reduced in meteoropathic individuals to prevent extremely dangerous situations [38, 39].

## DISCUSSION

This paper highlights the contemporary understanding of meteoropathy, which is no longer considered a popular myth, but a new disease. While previous studies have mainly focused on the impact of meteoropathy on individuals with mental illness, it is important to acknowledge that individuals with cardiovascular and respiratory diseases also experience an intensification of symptoms. However, the specific weather conditions that affect meteoropaths with different diseases may vary, necessitating the identification of weather conditions that impact each category of patients. Mobile applications that report temperature, wind velocity, wind direction, and barometric pressure can assist in this regard [26].

Considering that medical personnel can also be affected by meteoropathy, this should encourage us even more to extend our knowledge on how to prevent the aggravation of symptoms by monitoring the weather conditions. A healthy lifestyle, including a sleep regimen, hydration, preferably with water, a vitamin-rich diet, fresh fruits and vegetables, and physical activity (minimum 30 minutes daily, preferably walking), is highly recommended for meteoropathic patients. They should also stay in ventilated rooms and take hot and cold showers to help improve their circulation. Smoking and alcohol can increase body temperature and should be avoided [24, 40, 41].

We did not identify any studies on the role of genetics in meteoropathy or on the implication of different pharmacological pathways/ indicators in meteoropathy, which potentially may be considered as clinical markers and therapeutic targets. Currently, there is a gap in knowledge about specific pharmacological approaches to improve the quality of life of individuals with meteoropathy. However, considering the different types of meteoropathy and the clinical history of patients, we should work on developing individual pharmacological therapies for these patients.

Further research is also required on the role of naturopathy in meteoropathy. Meteropathic individuals should constantly check the weather forecast and take different supplements or herbs 48 hours before anticipated weather changes. For instance, chamomile and citrus tea can reduce tension, while Valeriana tea can reduce insomnia. Guarana seeds extract can enhance cognitive functions and decline anxiety. Siberian Ginseng extract also has a positive impact on meteoropaths. Lemon balm can improve cognitive functions, while lecithin and soy may enhance short- and long-term memory [42-44]. Other effects of herbs should also be studied in meteoropathy. Additionally, food supplements rich in Magnesium and Vitamin B-complex should also be taken. Homeopathy may also be effective in meteoropathy. Acupuncture, specifically auricular acupuncture, has been reported recently to reduce weather-induced symptoms induced by alterations in atmospheric barometric pressure [20].

## CONCLUSION

It is essential to recognize that meteoropathy is still not widely recognized in many countries, resulting in limited awareness of the diagnosis and alternative approaches to improving the quality of life for meteoropathic individuals. METEO-Q should be used to detect meteoropathy. Future research should not only focus on developing new individual therapies and identifying potential pharmacological targets through a clear understanding of the pathophysiology but also on raising awareness among the general population on the effects of weather changes on health and the importance of timely diagnosis and treatment. Establishing dedicated centers in various countries to study the impact of weather changes on health can facilitate the provision of timely weather forecasts to meteoropaths, alleviating their symptoms and improving their overall quality of life. Guarana seeds extract, chamomille, Siberian ginseng extract, lemon balm, lecithin, and soy, Valeriana tea are some herbs and supplements that may help improve the symptoms. Naturopathy and homeopathy should be considered as new approaches for offering alternative therapies to patients.
